# Utility of the Redox Cycle of Nitrofurantoin for the Development of a New Chemiluminescence Method for Its Analysis in Milk Samples

**DOI:** 10.3390/molecules30183698

**Published:** 2025-09-11

**Authors:** Mahmoud El-Maghrabey, Ali Abdel-Hakim, Shiho Tagaya, Naotaka Kuroda, Naoya Kishikawa

**Affiliations:** 1 Graduate School of Biomedical Sciences, Nagasaki University, 1-14 Bunkyo-machi, Nagasaki 852-8521, Japan; dr_m_hamed@mans.edu.eg (M.E.-M.); ali.hassan@fop.usc.edu.eg (A.A.-H.); tagaya.shiho76@chugai-pharm.co.jp (S.T.); n-kuro@nagasaki-u.ac.jp (N.K.); 2 Department of Pharmaceutical Analytical Chemistry, Faculty of Pharmacy, Mansoura University, Mansoura 35516, Egypt; 3 Department of Analytical Chemistry, Faculty of Pharmacy, University of Sadat City, Sadat City 32897, Egypt

**Keywords:** nitrofurantoin, growth promoter, food quality control, redox cycle, luminol, chemiluminescence, milk samples

## Abstract

Nitrofurantoin is utilized in various industries, including dairy, livestock, poultry, and aquaculture, as a growth promoter and antibacterial agent. Because prolonged use can cause mutagenesis and other side effects, many countries have prohibited its use in food-producing animals. In this work, we introduce a simple, rapid, and highly sensitive chemiluminescence (CL) approach for the quantitation of nitrofurantoin using its redox cycle activity. Nitrofurantoin is reduced to nitrofurantoin radicals by the reducing agent dithiothreitol, and reactive oxygen species (ROS) formed during the reoxidation process (superoxide anion radical) are detected by luminol CL. The CL conditions were optimized, including types of solvents, CL and reducing reagents, and their concentrations. The method was validated as per International Council for Harmonization (ICHQ2(R2)) guidelines, regarding linearity, detection and quantitation limits, accuracy, and precision. A good linearity with *r* = 0.9992 was obtained between the CL intensity and the nitrofurantoin concentration in the range of 4.0–400.0 ng/mL with a high sensitivity down to 1.15 ng/mL. The method was utilized to determine nitrofurantoin in milk samples, and a good recovery range was obtained (97.5–103.1%; RSD ≤ 4.4%); the results were comparable to the reported method, demonstrating the method’s reliability. Finally, the method demonstrated good practicality using a recently developed assessment tool.

## 1. Introduction

Nitrofurantoin ((*E*)-1-[(5-nitro-2-furyl) methylideneamino]imidazolidine-2,4-dione) is an antibiotic approved by the Food and Drug Administration (FDA) in 1953 and has been used to treat urinary tract infections [1,2]. It has also been used as a growth promoter and to treat bacterial infections in many industries, including dairy, livestock, poultry, and aquaculture [3,4,5]. The long-term use of nitrofurantoin can cause many side effects, including pulmonary fibrosis and hepatotoxicity [6]. Long-term studies using bacteria isolated from wastewater have shown that nitrofurantoin is mutagenic [7]; therefore, its use in food-producing animals is prohibited in many countries, including Australia, the United States, the Philippines, Thailand, Brazil, and Japan [8,9]. However, owing to its effectiveness and low cost, it is still used illegally in many countries and regions, especially in developing countries. Therefore, a highly sensitive and rapid quantitative method for nitrofurantoin is needed.

The methods published for the quantitative estimation of nitrofurantoin include high-performance liquid chromatography (HPLC) with ultraviolet (UV) detector (HPLC-UV) [10,11] ultra-high-performance liquid chromatography (UHPLC) with UV detector (UHPLC-UV) [12], UHPLC coupled with high-field quadrupole-Orbitrap high-resolution mass spectrometric detection (UHPLC-QE HF HRMS) [13], UV-visible spectrophotometry [14,15], fluorescence spectroscopy [16,17,18], optical methods [19,20], surface-enhanced Raman spectroscopy (SERS) [21,22,23], and voltammetry [24,25,26,27,28]. However, UV-visible spectrophotometry and optical methods generally exhibit poor sensitivities. Although SERS is a common quantitative method for nitrofurantoin owing to its high sensitivity and selectivity, it utilizes specific nanostructures prepared by complicated procedures. Chromatographic and voltammetric methods are highly sensitive and selective; however, they require complex and expensive equipment and time-consuming procedures. Fluorometric methods for nitrofurantoin analysis [16,17,18] have some drawbacks, such as the need for the preparation of carbon dots, and their detection mechanisms are based on the inner-filter effect, which affects their selectivity. In addition, they have poor sensitivity.

In this study, we focused on chemiluminescence (CL) as a method to rapidly and sensitively quantify nitrofurantoin, using relatively simple equipment. CL is a phenomenon in which an excited state molecule, generated by receiving energy from a chemical reaction, releases energy as light when it returns to a stable ground state [29]. The CL method does not need an excitation light source; therefore, it is possible to detect with high sensitivity without noise or scattered light from the light source, and it has the advantage of being able to measure with relatively simple equipment. Taokaenchan et al. [30] introduced a CL-based method for the estimation of nitrofurantoin. The technique used tris(2,2′-bipyridyl)ruthenium(II) as a CL reagent, but the method also used quantum dots capped with cysteine, which require a long synthesis time (more than 5 h), and the technique exhibited relatively poor sensitivity (0.6 μM). In another study, nitrofurantoin was determined by CL using a bis(2,4,6-trichlorophenyl) oxalate–H_2_O_2_ system [31]. However, this method requires the synthesis of a molecularly imprinted polymer (MIP), which complicates the procedure. Luminol is another CL reagent that emits intense light through reactive oxygen species (ROS) during the oxidation process and therefore has various applications [32]. The Luminol-H_2_O_2_ system was applied for post-column CL detection of nitrofurantoin [33]. However, chromatographic analysis followed by a post-column reaction makes this method more sophisticated.

**Figure 1:**
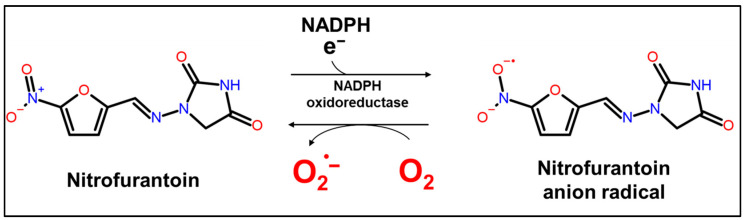
Illustration of the nitrofurantoin redox cycle.

Our laboratory has focused on analyzing quinones and their use as derivatizing agents in innovative and versatile ways [34,35,36,37]. We have developed a novel approach for quantitative CL analysis of quinones, in which they generate ROS [37,38] through a new CL reaction mechanism. Quinones are known to be reduced to radical species by the action of NADPH oxidoreductase, in vivo, by the donation of electrons from NADPH, which converts dissolved oxygen into ROS. Our CL reaction approach is based on the principle that dithiothreitol (DTT) is used as a reducing agent instead of NADPH to reduce quinones to radical species, and the ROS generated as a result is detected by CL with luminol. On the other hand, it has been reported that nitrofurantoin is reduced to radical species by the action of NADPH oxidoreductase and generates ROS, thereby oxidizing and decomposing bacteria and exerting an antibacterial effect [39] as illustrated in Figure 1. Therefore, we hypothesized that nitrofurantoin could also be quantitatively determined by CL using DTT and luminol as reagents, similar to quinones.

**Figure 2:**
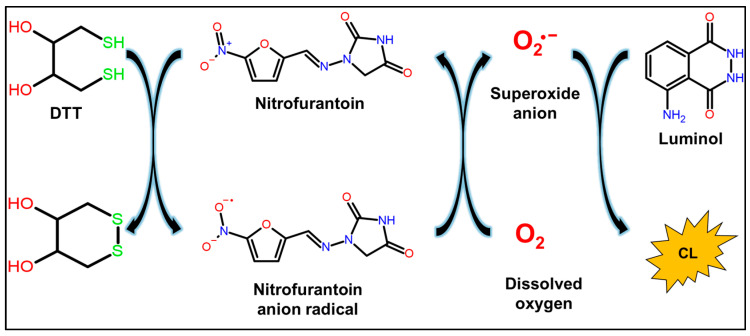
CL detection of nitrofurantoin based on its redox cycle.

Figure 2 shows an outline of the quantitative CL method that utilizes the redox cycle of nitrofurantoin, which is reduced by DTT to an unstable nitrofurantoin anion radical, which reacts with dissolved oxygen in the solution and is reoxidized, generating ROS. The ROS generated at this time can be detected by the CL using luminol, and the CL corresponding to the concentration of nitrofurantoin can be measured.

In this study, we optimized various reaction conditions, such as the type and concentration of the reagents, to establish a CL quantitative method for nitrofurantoin. Then, the method was validated and applied for the quantitative estimation of nitrofurantoin in milk samples, and the results obtained were compared with a reported method [17] to further prove the validity of the proposed method. Also, we identified the type of ROS involved in the CL using a selective ROS scavenger to confirm the mechanism of CL by nitrofurantoin.

## 2. Results and Discussion

### 2.1. CL Profile of Luminol/DTT/Nitrofurantoin System

**Figure 3:**
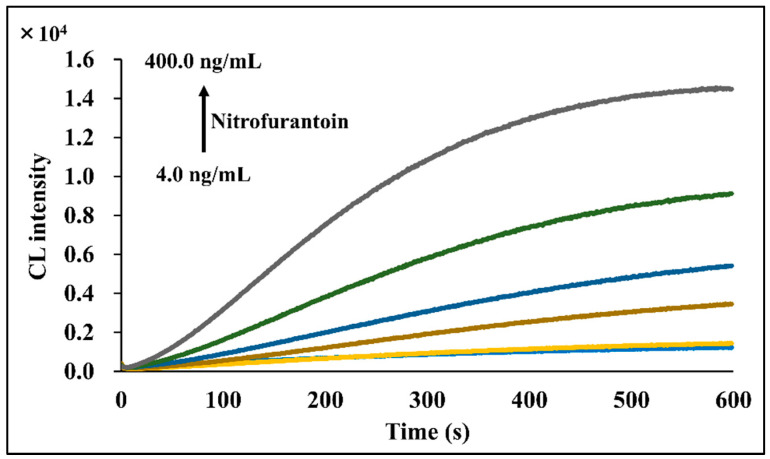
CL time profile for the reaction of luminol/DTT/nitrofurantoin system. [Conditions: 100 µL of 4.0–400.0 ng/mL nitrofurantoin in 15% ACN(*aq*), 100 µL of 500 µM luminol in 50 mM NaOH(*aq*), and 100 µL of 600 µM DTT in H_2_O].

The CL profiles of the luminol/DTT/nitrofurantoin system obtained by measuring nitrofurantoin solutions with different concentrations covering a range of 4.0–400.0 ng/mL under the established measurement operating conditions (described in Section 3.5) are shown in Figure 3. Stable CL was observed for more than 10 min immediately after the addition of DTT. Furthermore, the CL signal increased with increasing nitrofurantoin concentration, indicating its suitability for the quantitative determination of nitrofurantoin using the CL generated from its reaction with DTT and luminol.

### 2.2. Optimization of CL Reaction Conditions

Various CL reaction conditions were optimized, such as the type and concentration of the CL reagent, type and concentration of the reducing agent, acetonitrile (ACN) content in nitrofurantoin solution, and NaOH concentration in luminol solution.

#### 2.2.1. Selection of CL Reagent

**Figure 4:**
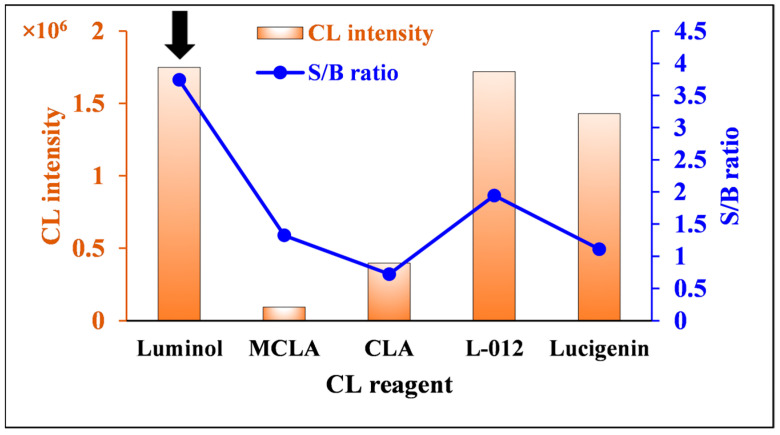
Effects of CL reagent on CL intensity and the S/B ratio of nitrofurantoin CL. [Conditions: 100 µL 80.0 ng/mL nitrofurantoin in 15% ACN(*aq*), 100 µL 500 µM CL reagent in 6 mM NaOH(*aq*), and 100 µL 600 µM DTT in H_2_O]. The black arrow indicates the optimum condition.

Five CL reagents were examined for detecting ROS generated by nitrofurantoin: luminol, luminol derivative L-012, lucigenin, 2-methyl-6-(4-methoxyphenyl)-3,7-dihydroimidazo [1,2-a]pyrazin-3-one (MCLA), and 2-methyl-6-phenyl-3,7-dihydroimidazo [1,2-a]pyrazin-3-one (CLA), which are analogs of Cypridina luciferin. CL measurements were performed three times, and the optimal conditions were evaluated by comparing the average values of the integrated CL and the signal/blank (S/B) ratio. The S/B ratio was calculated by dividing the integrated CL of the sample by the integrated CL of the blank. As shown in Figure 4, strong CL was obtained when lucigenin, L-012, and luminol were used as CL reagents, while the S/B ratio was maximized when luminol was used. Therefore, luminol was chosen as the optimal CL reagent in this work.

#### 2.2.2. Effect of Luminol Concentration

**Figure 5:**
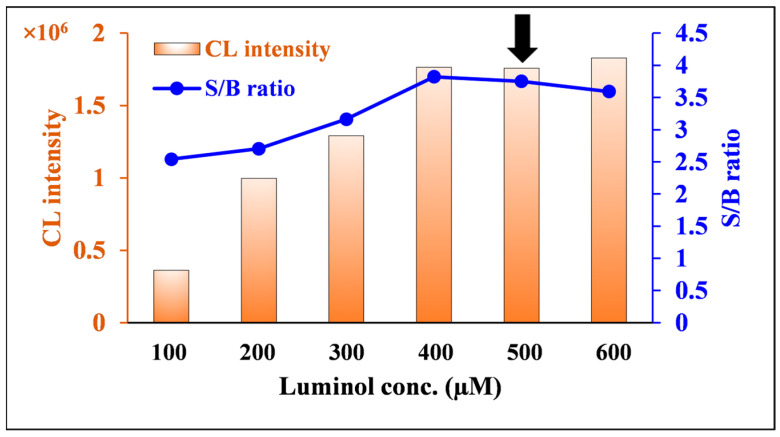
Effects of luminol concentration on CL intensity and S/B ratio of nitrofurantoin CL. [Conditions: 100 µL of 80.0 ng/mL nitrofurantoin in 15% ACN(*aq*), 100 µL of 100–600 µM luminol in 6 mM NaOH(*aq*), and 100 µL of 600 µM DTT in H_2_O]. The black arrow indicates the optimum condition.

The luminol concentration was investigated in the range of 100–600 µM. As shown in Figure 5, both the S/B ratio and integrated CL increased with increasing luminol concentration over a concentration range of 100–400 µM and reached a maximum and constant value at concentrations of 400 µM or more. Based on these results and taking robustness into consideration, 500 µM was chosen as the optimal luminol concentration.

#### 2.2.3. Effect of the Type of Reducing Agent

**Figure 6:**
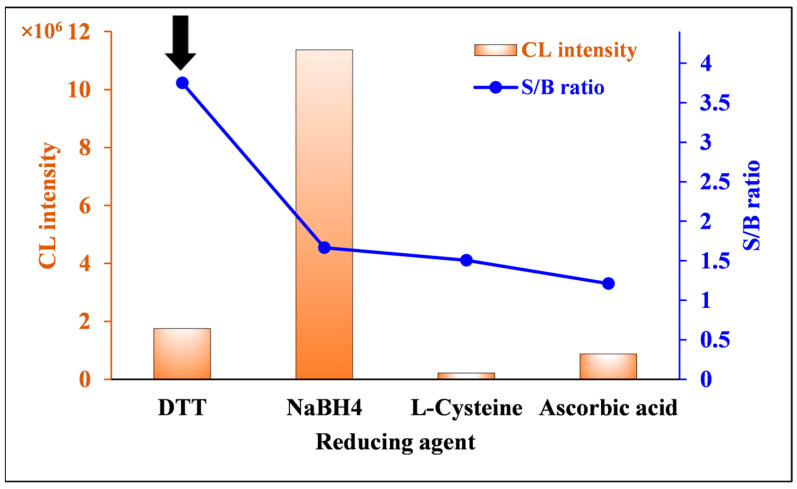
Effects of reducing agent on CL intensity and S/B ratio of nitrofurantoin CL. [Conditions: 100 µL of 80.0 ng/mL nitrofurantoin in 15% ACN(*aq*), 100 µL of 500 µM luminol in 6 mM NaOH(*aq*), and 100 µL of 600 µM reductants in H_2_O]. The black arrow indicates the optimum condition.

Four reducing agents, DTT, sodium borohydride (NaBH_4_), L-cysteine, and ascorbic acid, were used to reduce nitrofurantoin to radical species and compared. As shown in Figure 6, the maximum integrated CL was obtained when NaBH_4_ was used as the reducing agent, whereas the S/B ratio was the highest when DTT was used. Based on these results, DTT was selected as the optimal reducing agent for this study.

#### 2.2.4. Effect of DTT Concentration

**Figure 7:**
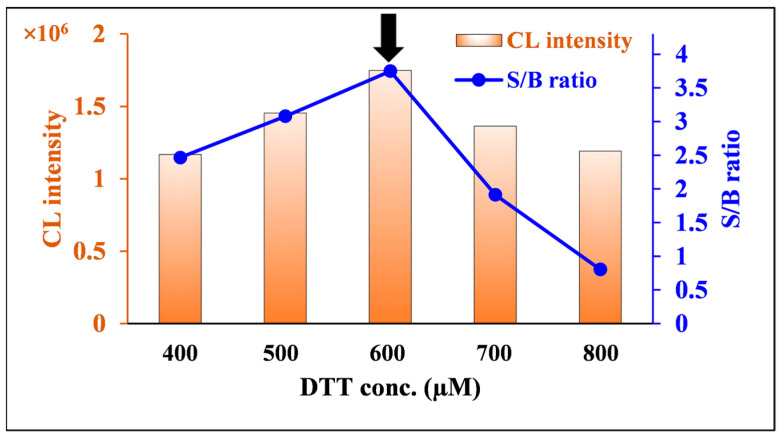
Effects of DTT concentration on CL intensity and the S/B ratio of nitrofurantoin CL. [Conditions: 100 µL of 80.0 ng/mL nitrofurantoin in 15% ACN(*aq*), 100 µL of 500 µM luminol in 6 mM NaOH(*aq*), and 100 µL of 400–800 µM DTT in H_2_O]. The black arrow indicates the optimum condition.

DTT concentration was investigated in the range of 400–800 µM. The maximum S/B ratio and integrated CL were achieved at 600 µM DTT (Figure 7). Therefore, 600 µM was chosen as the optimal DTT concentration for further experiments.

#### 2.2.5. Effect of ACN Content in Nitrofurantoin Solvent

**Figure 8:**
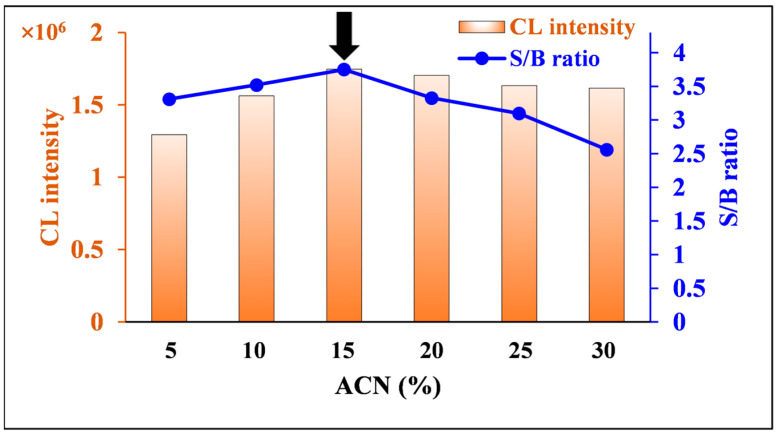
Effects of ACN content in nitrofurantoin solvent on CL intensity and S/B ratio of nitrofurantoin CL. [Conditions: 100 µL of 80.0 ng/mL nitrofurantoin in 5–30% ACN(*aq*), 100 µL of 500 µM luminol in 6 mM NaOH(*aq*), and 100 µL of 600 µM DTT in H_2_O]. The black arrow indicates the optimum condition.

The ACN content in the solvent used to prepare the nitrofurantoin solution was optimized in the range of 5–30%. The maximum S/B ratio and integrated CL were achieved with 15% ACN (Figure 8). Therefore, 15% ACN was chosen as the optimal ACN content for the nitrofurantoin solvent.

#### 2.2.6. Effect of NaOH Concentration

**Figure 9:**
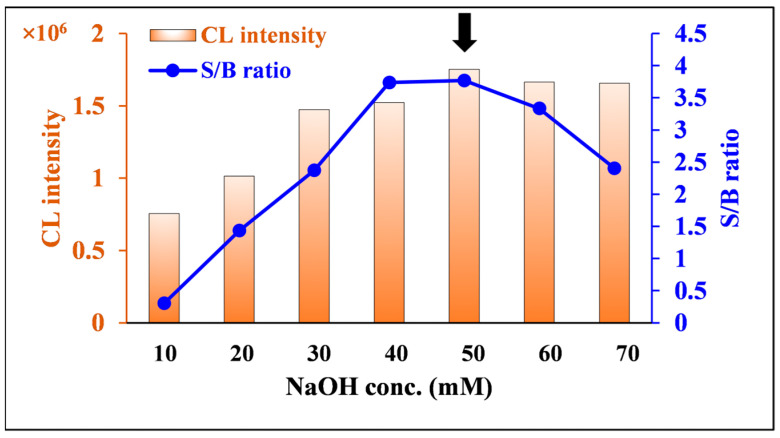
Effects of NaOH concentration on CL intensity and S/B ratio of nitrofurantoin CL. [Conditions: 100 µL 80.0 ng/mL nitrofurantoin in 15% ACN(*aq*), 100 µL 500 µM luminol in 10–70 mM NaOH(*aq*), and 100 µL 600 µM DTT in H_2_O]. The black arrow indicates the optimum condition.

Since luminol produces strong CL under alkaline conditions [40], the NaOH concentration in the solvent used to prepare the luminol solution was optimized in the range of 10–70 mM. As shown in Figure 9, the integrated CL and S/B ratio increased with increasing NaOH concentration. The integrated CL reached a nearly maximum and constant value at 40 mM or higher, and the S/B ratio slightly decreased at concentrations higher than 50 mM. Based on these results, 50 mM of NaOH was selected as the optimal concentration.

### 2.3. Identification of ROS Produced by CL of Nitrofurantoin

To elucidate the nitrofurantoin CL mechanism, the impact of selective ROS scavengers on CL produced by the three-component reaction of nitrofurantoin, DTT, and luminol was investigated in an attempt to identify the ROS that contribute to CL.

The quenching effect of selective ROS scavengers on CL generated by the reaction of nitrofurantoin, DTT, and luminol was examined. Superoxide dismutase (SOD) (1, 10 U/mL) was used as a selective ROS scavenger to quench O_2_**^·−^**, mannitol (10, 100 µM) and methanol (1, 10%) for ^•^OH, and sodium azide (NaN_3_) (10, 100 µM) for ^1^O_2_ [37,41]. As shown in Table 1, a notable decrease in the CL was observed with the addition of SOD. However, the quenching effect was not very high when ROS scavengers such as mannitol, methanol, and sodium azide were added. Therefore, it was suggested that the main ROS involved in the CL of nitrofurantoin was O_2_**^·−^**. This result is consistent with a previous report that nitrofurantoin exerts its bactericidal effect by generating O_2_^·−^ through an oxidation-reduction reaction in vivo [39].

### 2.4. Method Validation

The proposed approach was validated according to the International Council for Harmonization (ICHQ2(R2)) guidelines for validation of analytical procedures [42]. The calibration curve of the nitrofurantoin standard solution was prepared under optimal conditions. A good linear relationship was obtained between the nitrofurantoin concentration and integrated CL intensity, with a correlation coefficient of 0.9992 in the concentration range of 4.0–400.0 ng/mL. The limit of detection (LOD) and limit of quantitation (LOQ) were calculated using the standard deviation (S.D.) of the blank and slope of the calibration curve. The LOD and LOQ of this method were 1.15 ng/mL and 3.45 ng/mL, respectively. The accuracy and precision of the proposed CL method were assessed using five nitrofurantoin levels within the method’s linear range. The % recovery of triplicate analysis of each concentration ranged from 98.0 to 102.5%, indicating the excellent accuracy of the developed method. The relative standard deviation (RSD) was not more than 5.5% for intra-day precision, and 8.6% for inter-day precision, indicating the good precision of the proposed CL method. The results of the accuracy and precision of the method are presented in Table 2.

The sensitivity of this method for nitrofurantoin was compared with that of conventional quantitative methods, as shown in Table 3. The sensitivity of this CL method was 8.7–973 times higher than that of chromatographic methods with UV detectors [10,11,12], 141 times higher than that of the UV-visible spectrophotometric method [15], 8.7–289 times higher than that of the fluorescence spectroscopic methods [16,17,18], 18.6–173 times higher than that of the optical methods [19,20], 4.3–43.4 times higher than that of the SERS methods [21,22,23], and 1.7–9.9 times higher than that of the voltammetric methods [24,25,26,27,28]. Compared with the mass spectrometric-based method [13], this CL method was less sensitive than the mass spectrometric-based method. Although the sensitivity of the CL method is inferior, considering the measurement time and procedures required for one analysis, the CL method is considered superior in terms of simplicity and speed. This is because the developed CL method utilizes simple and cost-effective equipment (luminometer) and avoids the consumption of highly toxic organic solvents and the high energy associated with chromatographic analysis and MS detection. In addition, the mass spectrometric-based method uses a metal–organic framework, which is prepared in more than 12 h, making the method sophisticated and time-consuming. Therefore, the developed CL method is deemed to be more suitable for rapid analysis, particularly in resource-limited settings.

### 2.5. Application of the Developed Method for the Determination of Nitrofurantoin in Milk Samples

The developed CL method was used to analyze milk samples. First, non-spiked samples were assayed, and nitrofurantoin was absent from these samples. This is mostly because veterinary use of nitrofurantoin is banned in Japan, as mentioned earlier. Analysis of the non-spiked samples using the reported method also yielded the same results. Therefore, spiked samples were analyzed to evaluate the applicability of the method. As shown in Table 4, the proposed method achieved excellent recovery of nitrofurantoin from milk, ranging from 97.5% to 103.1%, indicating its applicability for determining nitrofurantoin in milk samples. Furthermore, the precision of the repeated measurements (*n* = 5) was calculated, and the RSD was not more than 4.4%, which demonstrates the excellent reliability of the developed method. To further confirm the validity of the developed method, its outcomes were statistically compared to the outcomes of the reported method [17], using Student’s *t*-test and *F*-test for variance ratio. As shown in Table 4, the calculated *t* and *F* values were lower than the tabulated values, indicating the absence of significant differences between the developed and published methods in terms of accuracy and precision.

### 2.6. Evaluation of the Method’s Practicability

Recently, a new tool named the Blue Applicability Grade Index (BAGI) was established to evaluate the practicability (blueness) of analytical methodologies. This method considers 10 attributes: the type of analysis, number of analytes concurrently assayed, instrumentation, number of samples simultaneously prepared, procedure for preparing the samples, number of samples analyzed in one hour, type of chemicals used, need for preconcentration, and sample quantity [43]. A score was assigned to each attribute. The lowest score is 2.5, and the highest score is 10. The lowest overall score is 25, and the highest overall score is 100. The overall score is the summation of all scores for all attributes and is determined by the tool’s software. The software also generates an asteroid graph, where each attribute is given a color (dark blue, the optimum, moderate blue, light blue, or white, the worst). For the analytical method to be considered practical, it must achieve an overall score of at least 60. Table 5 presents the blueness assessment of the developed method using the BAGI approach. The proposed method achieved a final score of 67.5, demonstrating good practicality.

## 3. Materials and Methods

### 3.1. Materials and Reagents

Luminol, DTT, sodium hydroxide (NaOH), L-cysteine, and chloroform were provided by Nacalai Tesque, Inc. (Kyoto, Japan). Nitrofurantoin, lucigenin, NaBH_4_, MCLA, and CLA were provided by Tokyo Chemical Industry Co., Ltd. (Tokyo, Japan). L-012, ascorbic acid, trichloroacetic acid, SOD, and sodium azide were purchased from Fujifilm Wako Pure Chemical Co., Ltd. (Osaka, Japan). Methanol and ACN were purchased from Kanto Chemical Co., Ltd. (Tokyo, Japan). Mannitol was provided by Sigma-Aldrich (St. Louis, MO, USA).

Luminal solution was prepared using an aqueous NaOH solution. Nitrofurantoin solution was prepared using an ACN aqueous solution and diluted to the desired concentration.

### 3.2. Equipment and Software

All the CL measurements were conducted using a Lumat LB-9507 luminometer (Berthold Technologies, Bad Wildbad, Germany), with a total integration time of 600 s, using 12 × 75 mm round-bottom test tubes (Fisher Scientific, Pittsburgh, PA, USA).

An ultrasonic cleaner, SND US-102 (Nagano, Japan), a Himac CR refrigerated centrifuge (Hitachi, Tokyo, Japan), and an automatic mixer S-100 (Taiyo Kagaku Kogyo Co., Ltd., Tokyo, Japan) were used for sonication, centrifugation, and mixing, respectively.

Yamato Autosill WG220 water purification device (Yamato Scientific Co., Ltd., Tokyo, Japan) was used to obtain the purified water.

Terumo syringes (1 mL, Terumo Medical Corp., Tokyo, Japan) fitted with hydrophilic PTFE 0.45 µm FILSTAR syringe filters (Starlab Scientific Co., Ltd., Beijing, China) were used for the preparation of milk samples.

Blueness assessment of the developed CL method by the BAGI tool was performed using the freely available web version of the tool at (https://bagi-index.anvil.app/, accessed on 25 August 2025), by selecting the most appropriate choice from the drop-down list of each evaluated parameter. Then, the generated graph was saved as a .png image. The chemical structures in Figure 1 and Figure 2 were generated using ACD/ChemSketch (freeware) version 2025.1.0.

### 3.3. Procedure for Optimization of CL Conditions

The CL conditions were optimized using a one-factor-at-a-time approach, in which one factor was studied at a time, while the other factors remained constant. The studied factors were the type and concentration of the CL reagent, the type and concentration of the reducing agent, ACN content in the nitrofurantoin solution, and NaOH concentration in the luminol solution. The following conditions were used unless stated otherwise (100 µL of 80.0 ng/mL nitrofurantoin in 15% ACN(*aq*), 100 µL of 500 µM luminol in 50 mM NaOH(*aq*), 100 µL of 600 µM DTT in H_2_O, and a total integration time of 600 s). In all cases, a blank experiment was conducted concurrently. Blank samples were prepared using the same procedure but with the replacement of the nitrofurantoin solution with 100 µL of 15% ACN(*aq*). The integrated CL and S/B ratios were used to determine the optimum conditions.

### 3.4. Procedure for Identification of ROS Responsible for the CL of Nitrofurantoin

One hundred microliters of luminol (500 µM in 50 mM NaOH solution), nitrofurantoin (40.0 ng/mL in 15% ACN), and an aqueous solution of ROS scavengers were added to a test tube and stirred for 5 s. The test tube was placed in a luminometer, followed by the addition of 100 µL of 600 µM aqueous solution of DTT, and the CL generated was measured for 600 s. The CL measurement was repeated three times.

### 3.5. Procedure for Method Validation

The developed method was validated following ICHQ2(R2) guidelines [42]. To construct the calibration curve, 100 µL of 500 µM luminol in 50 mM NaOH solution and 100 µL of 15% ACN solution with different concentrations of nitrofurantoin (to obtain a final concentration range of 4.0–400.0 ng/mL) were added to a series of test tubes and stirred for 5 s. The tubes were placed in a luminometer, and 100 µL of 600 µM DTT solution was added, and the resulting CL was measured for 600 s. CL measurements were repeated three times. The integrated CL values were plotted against the nitrofurantoin concentration (ng/mL) to construct a calibration curve. LOD and LOQ were estimated as LOD = 3.3 S.D. of blank/slope of the calibration curve, and LOQ = 10 S.D. of blank/slope of the calibration curve. Accuracy and precision were evaluated at five nitrofurantoin levels (8.0, 40.0, 80.0, 160.0, and 400.0 ng/mL). Each concentration was analyzed three times, and the average % recovery was determined using the regression equation to evaluate the accuracy of the method. The precision was evaluated by determining the %RSD for each concentration. The experiments were conducted on the same day (intra-day precision) and on three successive days (inter-day precision).

### 3.6. Determination of Nitrofurantoin in Milk Samples

The method reported by Liu et al. [44] was used for the extraction of nitrofurantoin from milk, with some modifications. Non-spiked milk samples were prepared by adding 800 µL of 10% trichloroacetic acid solution to 200 µL of the milk samples to remove proteins. Then, 1 mL of chloroform was added, and the mixture was shaken for 1 min and ultrasonicated for 20 min. The mixture was then centrifuged for 10 min, and the upper aqueous layer was collected. This upper layer was neutralized with 0.5 M NaOH aqueous solution, filtered through a membrane filter, diluted 10 times using 15% ACN solution, and used for CL measurement.

Spiked milk samples were prepared by adding different concentrations of nitrofurantoin (to obtain final concentrations of 40.0, 80.0, and 400.0 ng/mL) to 200 µL of milk samples, followed by the addition of 800 µL of a 10% trichloroacetic acid solution to remove proteins. They were then completed using the same steps as those used for the non-spiked samples.

## 4. Conclusions

In this study, the redox cycle of nitrofurantoin was utilized to develop a CL method for its analysis for the first time, where ROS are produced upon a reduction of nitrofurantoin by DTT, and the generated ROS reacted with luminol to produce CL. This method is simple, reliable, and cost-effective. It showed good linearity, sensitivity, accuracy, and precision, in addition to good applicability to milk samples, which is a very complicated matrix. The method’s excellent sensitivity was superior to that of most reported analytical methods for the estimation of nitrofurantoin. In addition, the method showed a good blueness profile, further indicating its applicability to real sample analysis. Future investigations should focus on expanding the scope of the application of the developed method beyond milk samples to include other food matrices and biological fluids. In addition, using a selective extraction approach such as MIP or integration of chromatographic separation prior to CL detection would resolve possible interference and allow for further broadening the scope of the method. Although this is deemed unnecessary in the analysis of milk samples by the developed method, it might be helpful if other compounds capable of redox cycling or generating ROS are present in other food matrices or biological fluids being investigated.

## Figures and Tables

**Table 1:** Effect of selective ROS scavengers on CL production by nitrofurantoin, DTT, and luminol.

Scavenger	ROS	Concentration	RCI *
No scavenger	–	–	100
SOD	O_2_^·−^	1 U/mL	0.61
		10 U/mL	0.25
Mannitol	^•^OH	10 μM	56.4
		100 μM	47.9
Methanol	^•^OH	1%	71.1
		10%	52.8
NaN_3_	^1^O_2_	10 μM	63.6
		100 μM	53.7

* RCI refers to relative CL intensity. The value was taken as 100 for nitrofurantoin with luminol and DTT without a scavenger.

**Table 2:** Results of the accuracy and precision study of nitrofurantoin using the proposed CL method.

Concentration Added (ng/mL)	Recovery (%)	Intra-Day Precision (RSD, %)	Inter-Day Precision (RSD, %)
8.0	102.5	3.3	8.6
40.0	98.0	1.4	2.4
80.0	99.5	5.5	6.2
160.0	99.8	5.5	6.0
400.0	101.3	1.2	1.8

**Table 3:** Comparison of the sensitivity of the proposed method with previously reported methods.

Method	Range (ng/mL)	LOD (ng/mL)	Ref.
HPLC-UV	10,000–100,000	1119	[10]
HPLC-UV	200–20,000	10	[11]
UHPLC-UV	50–1250	27	[12]
UHPLC-QE HF HRMS	1–100	0.3	[13]
UV-visible spectrophotometry	5000–25,000	–	[14]
UV-visible spectrophotometry	500–30,000	163	[15]
Fluorescence spectroscopy	11,900–21,420	333	[16]
Fluorescence spectroscopy	500–8000	140	[17]
Fluorescence spectroscopy	570–28,580	10	[18]
Optical detection	200–19,040	200	[19]
Optical detection	21.4–38,080	21.4	[20]
SERS	500–10,000	50	[21]
SERS	50–1000	14	[22]
SERS	5–500	5	[23]
Voltammetry	12–54,740	2.4	[24]
Voltammetry	3.6–59,262	3.6	[25]
Voltammetry	12–130,900	4.4	[26]
Voltammetry	8.3–160,007	2	[27]
Voltammetry	119–28,560	11.4	[28]
CL	4.0–400.0	1.15	This method

**Table 4:** Determination of nitrofurantoin in milk samples using the proposed CL method and the reported method.

Proposed Method			Reported Method [17]	
Concentration Added (ng/mL)	Recovery (%)	Precision (RSD, %)	Concentration Added (ng/mL)	Recovery (%)
0	Not detected	–	0	Not detected
40.0	103.1	2.3	500.0	96.4
80.0	99.5	3.5	1000.0	102.3
400.0	97.5	4.4	1500.0	95.7
Mean recovery ± S.D.	100.03 ± 2.83		98.13 ± 3.62	
*t*-test (2.776) *	0.71			
*F*-test (19.00) *	1.63			

* The values in parentheses are the tabulated *t* and *F* values at *p* = 0.05.

**Table 5:** Evaluation of the practicability of the proposed method using the BAGI tool.

Attribute	Result	Color	Pictogram
Type of analysis	Quantitative	Moderate blue	
2. Analyte number	Single element	While	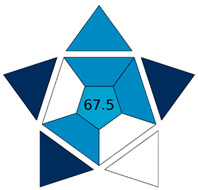
3. Analytical technique	Simple instruments	Moderate blue	
4. Simultaneous sample preparation	1	White	
5. Sample preparation	Simple, low-cost sample preparation required	Moderate blue	
6. Samples per hour	5–10	Moderate blue	
7. Reagents and materials	Common commercially available reagents	Dark blue	
8. Preconcentration	Not needed	Dark blue	
9. Degree of automation	Manual treatment and analysis	White	
10. Sample quantity	Less than 10 mL of the food sample	Dark blue	
Overall score		67.5	

## Data Availability

The original contributions presented in this study are included in the article. Further inquiries can be directed to the corresponding author.

## Abbreviations

The following abbreviations are used in this manuscript:

CL	Chemiluminescence
ROS	Reactive Oxygen Species
ICH	International Council for Harmonization
FDA	Food and Drug Administration
HPLC	High-Performance Liquid Chromatography
UHPLC	Ultra-High-Performance Liquid Chromatography
UV	Ultraviolet
QE HF HRMS	High-Field Quadrupole-Orbitrap High Resolution Mass Spectrometry
SERS	Surface-Enhanced Raman Spectroscopy
MIP	Molecularly Imprinted Polymer
DTT	Dithiothreitol
ACN	Acetonitrile
MCLA	2-methyl-6-(4-methoxyphenyl)-3,7-dihydroimidazo [1,2-a]pyrazin-3-one
CLA	2-methyl-6-phenyl-3,7-dihydroimidazo [1,2-a]pyrazin-3-one
S/B	Signal to Blank Ration
SOD	Superoxide dismutase
RCI	Relative CL Intensity
LOD	Limit of Detection
LOQ	Limit of Quantitation
S.D.	Standard Deviation
RSD	Relative Standard Deviation
BAGI	Blue Applicability Grade Index
